# Molecular characterization of siderophore biosynthesis in *Paracoccidioide*s *brasiliensis*

**DOI:** 10.1186/s43008-020-00035-x

**Published:** 2020-06-29

**Authors:** Marielle Garcia Silva, Juliana Santana de Curcio, Mirelle Garcia Silva-Bailão, Raisa Melo Lima, Mariana Vieira Tomazett, Aparecido Ferreira de Souza, Vanessa Rafaela Milhomem Cruz-Leite, Nicolau Sbaraini, Alexandre Melo Bailão, Fernando Rodrigues, Maristela Pereira, Relber Aguiar Gonçales, Célia Maria de Almeida Soares

**Affiliations:** 1 Laboratório de Biologia Molecular, Instituto de Ciências Biológicas, ICB II, Campus II, Universidade Federal de Goiás, Goiânia, GO Brazil; 2 Programa de Pós-graduação em Patologia Molecular, Faculdade de Medicina, Universidade de Brasília, Brasília, DF 70910-900 Brazil; 3 Centro de Biotecnologia, Programa de Pós-graduação em Biologia Celular e Molecular, Universidade Federal do Rio Grande do Sul, Porto Alegre, RS Brazil; 4 Life and Health Sciences Research Institute (ICVS), School of Medicine, University of Minho, Braga, Portugal; 5 ICVS/3B’s - PT Government Associate Laboratory, Braga/Guimarães, Portugal

**Keywords:** Iron, SidA, RNA interference

## Abstract

Iron is an essential nutrient for all organisms. For pathogenic fungi, iron is essential for the success of infection. Thus, these organisms have developed high affinity iron uptake mechanisms to deal with metal deprivation imposed by the host. Siderophore production is one of the mechanisms that fungal pathogens employ for iron acquisition. *Paracoccidioides* spp. present orthologous genes encoding the enzymes necessary for the biosynthesis of hydroxamates, and plasma membrane proteins related to the transport of these molecules. All these genes are induced in iron deprivation. In addition, it has been observed that *Paracoccidioides* spp. are able to use siderophores to scavenge iron. Here we observed that addition of the xenosiderophore ferrioxamine B FOB) to *P. brasiliensis* culture medium results in repression (at RNA and protein levels) of the SidA, the first enzyme of the siderophore biosynthesis pathway. Furthermore, SidA activity was reduced in the presence of FOB, suggesting that *P. brasiliensis* blocks siderophores biosynthesis and can explore siderophores in the environment to scavenge iron. In order to support the importance of siderophores on *Paracoccidioides* sp. life and infection cycle, silenced mutants for the *sidA* gene were obtained by antisense RNA technology. The obtained *AsSidA* strains displayed decreased siderophore biosynthesis in iron deprivation conditions and reduced virulence to an invertebrate model.

## INTRODUCTION

Iron is an essential nutrient for growth and development of living organisms. Due to iron redox properties, this metal occurs in two oxidation states, ferrous ion (Fe^+ 2^) and ferric ion (Fe^+ 3^), which are influenced by pH and oxygen (Sanchez et al. 2017). Fe^+ 2^ spontaneously convert to Fe^+ 3^ in the presence of oxygen (Halliwell and Gutteridge 1984). Iron is indispensable for a variety of cellular processes such as respiration, although, the excess or incorrect storage of this metal by cells is harmful. The reduced form of iron (Fe^+ 2^) catalyzes the production of reactive oxygen species (ROS) through the Fenton/Haber Weiss reaction (Halliwell and Gutteridge 1984; Haber and Weiss 1934). In this way, the maintenance of the homeostasis of this micronutrient is essential. Proper iron homeostasis is achieved through fine-tuned regulation of iron acquisition, use and storage (Ganz 2009).

Nutritional immunity (i.e., host deprivation of metals, such Fe, Cu, Mn and Zn) is an important mechanism employed by the host to control the development of pathogenic organisms, to overcome the low availability of iron imposed by the host, microorganisms developed high affinity mechanisms for iron uptake (Raymond et al. 2003). In fungal pathogens these mechanisms include: the reduction of Fe^+ 3^ to Fe^+ 2^, the acquisition of the iron bound to the heme group, and the solubilization of Fe^+ 3^ promoted by siderophores (Kornitzer 2009; Bailão et al. 2012; Canessa and Larrondo 2013). The last strategy, also known as non-reductive iron uptake, is characterized by the use of siderophores, which are low molecular weight compounds that bind Fe^+ 3^ with high affinity making it available for consumption (Neilands 1993).

Fungal siderophore biosynthesis is well characterized in *Aspergillus fumigatus* (Blatzer et al. 2011; Schrettl et al. 2004; Schrettl et al. 2007) and internalization of the siderophore-iron complex is performed by Siderophore Iron Transporters (SIT), such as Sit, MirA, MirB and MirC, located on the cell surface, as described in *Candida glabrata* and *Aspergillus nidulans* (Nevitt and Thiele 2011; Haas 2003; Haas et al. 2003). Siderophores are also produced by fungi of the *Paracoccidioides* genus that cause paracoccidioidomycosis (PCM) (Restrepo 1985), a disease restricted to Latin America (San-Blas et al. 2002) with high rates in Brazil (Restrepo et al. 2001). *Paracoccidioides* spp. grow as mycelia in the environment and as yeast cells in host tissues (Restrepo 1985). After inhalation of conidia or mycelial propagules, these reach the pulmonary alveoli of the host and differentiate into yeast cells, thus initiating the infectious process (McEwen et al. 1987).

Fungi of the *Paracoccidioides* genus can use reductive (Fe^+ 3^ to Fe^+ 2^) and siderophore uptake pathways to acquire iron under conditions of metal shortage (Silva et al. 2011; Silva-Bailao et al. 2014; Bailao et al. 2015). We have demonstrated that *Paracoccidioides* spp. present putative orthologue genes to those related to hydroxamate siderophore production (*sidA*, *sidF*, *sidC*, *sidD, SidH* and *sidI*) as well as siderophore uptake (*sit1*, *mirB* and *mirC*) (Silva et al. 2011; Silva-Bailao et al. 2014). All of them are up-regulated in iron restriction and, in such condition, hydroxamate siderophores are produced (Silva-Bailao et al. 2014, Parente et al. 2011). Furthermore, *Paracoccidioides* spp. can also explore siderophores from other organisms to scavenge iron, as dimerumic acid and ferrioxamine B (FOB). Additionally, prior exposure of *P. brasiliensis* to FOB increases fungus survival to phagocytosis by activated macrophages (Silva-Bailao et al. 2014).

Considering those findings, we sought to investigate the adaptation of *P. brasiliensis* after FOB exposure as well as the functional role of SidA, the first enzyme in the siderophore production, in this fungus. Moreover, knockdown strains for *sidA* were generated employing antisense RNA technology and *Agrobacterium tumefaciens*-mediated transformation (ATMT) (Almeida et al. 2007; Menino et al. 2012; Bailao et al. 2014). Notably, upon FOB exposure, *P. brasiliensis’ sidA* was down regulated at transcriptional and translational levels, which was accompanied by reduced enzymatic activity. Furthermore, the knockdown of *sidA* (*AsSidA*) led to reduced siderophore production by *P. brasiliensis* and decreased fungal virulence to *Tenebrio molitor* an invertebrate model, suggesting an essential role of siderophores in the infection cycle of *P. brasiliensis*.

## MATERIAL AND METHODS

### Ethics statement

Mouse manipulation was carried out in accordance with the ethical principles of animal research adopted by the Brazilian Society of Laboratory Animal Science and a Brazilian Federal Law 11.749 (October 2008). Male BALB/c mice aged between 6 to 8 weeks were purchased from the Animal house of the Instituto de Patologia Tropical e Saúde Pública – UFG and were maintained in the Animal Facilities at the Laboratório de Biologia Molecular, Universidade Federal de Goiás. Animal experimentation was approved by institutional Ethics Commission on Animal Use of the Universidade Federal de Goiás – UFG (reference number 089/17).

### Strains and culture conditions

Yeast cells of *P. brasiliensis*, *Pb*18 (ATCC32069) were used in all the experiments. Cells were maintained in brain heart infusion (BHI) solid medium added of 4% (w/v) glucose for 4 days, at 36 °C. For experiments, cells were grown in liquid BHI for 72 h at 36 °C, 150 rpm, in order to reach the exponential growth phase (10^7^ cells per ml). Afterward, the cells were centrifuged at 1200 x *g* for 10 min at 4 °C and washed twice with Phosphate Buffered Saline (PBS) 1X. Cells were then incubated in McVeigh/Morton liquid medium (MMcM) (Restrepo and Jimenez 1980) containing: 4% (w/v) glucose, 0.15% (w/v) KH_2_PO_4_, 0.05% (w/v) MgSO_4_.7H_2_O, 0.015% (w/v) CaCl_2_.2H_2_O, 0.2% (w/v) (NH_4_)_2_SO_4_, 0.2% (w/v) L-asparagine, 0.02% (w/v) L-cystine, 1% (v/v) of vitamin supplement (0.006% [w/v] thiamine, 0.006% [w/v] niacin B3, 0.006% [w/v] Ca^+ 2^ pantothenate, 0.001% [w/v] inositol B7, 0.0001% [w/v] biotin B8, 0.001% [w/v] riboflavin, 0.01% [w/v] folic acid B9, 0.01% [w/v] choline chloride, 0.01% [w/v] pyridoxine) and 0.1% (v/v) of trace elements supplement (0.0057% [w/v] H_3_BO_3_, 0.0081% [w/v] MnSO_4_.14H_2_O, 0.0036% [w/v] (NH_4_)_6_MO_7_O_24_.4H_2_O, 0.0157% [w/v] CuSO_4_.H_2_O, 0.1404% [w/v] Fe(NH_4_)_2_(SO_4_)_2._6H_2_O) (Restrepo and Jimenez 1980) supplemented with 50 μM of bathophenanthroline-disulfonic acid (BPS; Sigma-Aldrich, Germany), a ferrous iron-specific chelator, for 24 h at 36 °C with shaking at 150 rpm (Parente et al. 2011, Silva-Bailao et al. 2014). Cells were centrifuged and washed twice with PBS 1X and cell viability was determined using trypan blue. A total of 10^7^ cells per mL were transferred to MMcM medium containing 50 μM of BPS or 10 μM of ferrioxamine B with iron loaded (FOB; Sigma-Aldrich, Saint louis, USA) (Silva-Bailao et al. 2014). Yeast cells were incubated at 36 °C for 6 and 24 h, 150 rpm. For culture in MMcM medium all the glassware was acid treated to remove residual traces of iron (Cox 1994).

### RNA extraction and quantitative real time PCR (RT-qPCR)

Total RNA extraction was accomplished using TRIzol (TRI Reagent, Sigma-Aldrich, St. Louis, MO) and mechanical cell rupture (Mini-Beadbeater – Biospec Products Inc., Bartlesville, OK). The mRNA was reverse-transcribed using Super-Script III First-Strand Synthesis SuperMix (Invitrogen, Life Technologies). qRT-PCR was performed employing a QuantStudio5 real-time PCR system (Applied Biosystems Inc.) and SYBER green PCR master mix was used in the reaction mixture (Applied Biosystems, Foster City, CA). The sequences of forward and reverse oligonucleotides are listed in (Additional file 1: Table S1). The data were normalized with the 28 kDa ribonucleoprotein (GenBank accession number XP_015701336). The relative expression levels of transcripts of interest were calculated using the standard curve method for relative quantification (Bookout et al. 2006).

### L-ornithine-N^5^-oxygenase enzymatic assay

The enzymatic activity of L-ornithine-N^5^-oxygenase (SidA) was evaluated as previously described by Zhou et al. (1998) and Haas et al. (1999) with few modifications. Briefly, *P. brasiliensis* yeast cells were collected and suspended in 0.5 mM potassium phosphate buffer (pH 8.0). The suspension was transferred to tubes containing glass beads (425–600 μm) and submitted to vigorous mixing in a bead beater apparatus (BioSpec) for 5 cycles with intervals of 30 s on ice. The samples were centrifuged 10,000 x *g* for 15 min and the protein concentration in supernatants was determined with the (Bradford) reagent. To measure the enzymatic activity of SidA, equal amounts of proteins (50 μg) of both conditions, yeast cells incubated with (BPS) or (FOB), were used. The reaction mixture containing 40 μl of 0.5 mM potassium phosphate pH 8.0, 10 μl of 10 mM NADPH, 2 μl of 0.5 mM FAD, 50 μg of cell extract, 30 μl of 10 mM L-ornithine was incubated at 30 °C for 2 h and added of 100 μl of 0.2 M perchloric acid to stop the reaction. For control, the same amount of perchloric acid was added to one sample before incubation. The samples were centrifuged and the supernatants were used to determine the absorbance at 340 nm.

### Molecular modeling of SidA

The amino acid sequence of the *P. brasiliensis* SidA (PADG_00097) was modeled with ITASSER algorithm (Yang and Zhang 2015) available at (https://zhanglab.ccmb.med.umich.edu/). To predict protonation states of the model, the PDB2PQR server (http://nbcr-222.ucsd.edu/pdb2pqr_2.0.0/) at pH 7 was used. Pymol visualizer was used to perform the structural analysis (Rigsby and Parker 2016).

The MD simulation was performed by the GROMACS 4.5.5 package, using the AMBER force field (ff99SB-ILDM) in the presence of water TIP3P. The protein was subjected to the simulation of 100 ns, temperature of 300 K, pressure of 1 atm and time interval of 2 fentoseconds, without restriction of the conformation (Pronk et al. 2013).

Analysis of Clusters, Root Mean Square Deviation (RMSD) and Root Mean Square Fluctuations (RMSF) were performed using the software of the GROMACS package. The quality analysis and the Ramachandran diagram of the final MD model were performed using the MolProbity server (http://molprobity.biochem.duke.edu/) (Chen et al. 2010).

### Heterologous expression of recombinant SidA

Primers used for amplification of *SidA* cDNA were listed in (Additional file 1: Table S1). The PCR product was sub cloned into the *Bam*HI*/Eco*RI sites of pGEX-4 T3 vector (GE Healthcare Life Sciences). Transformation of *Escherichia coli* Rosetta (DE3) was carried out using standard procedures. For protein expression, transformed cells were cultured in LB medium supplemented with ampicillin (100 μg/ml) for 16 h at 37 °C. The induction of the recombinant protein was performed by addition of Isopropyl β-D-1-thiogalactopyranoside (IPTG; Sigma-Aldrich, St Louis, MO, USA) at a final concentration of 1 mM for 2 h. The size and identity of the recombinant protein SidA (rSidA) was evaluated using SDS-PAGE and in-gel protein digestion (Rezende et al. 2011) followed by LC-MS/MS (Lima Pde et al. 2015).

### Polyclonal antibodies production and immunoblotting assay

rSidA was used in the production of specific mouse polyclonal antibodies. Pre-immune sera were obtained and stored at − 20 °C. The rSidA was extracted from the SDS-PAGE polyacrylamide gel, and subsequently injected into mouse three times at 15 days intervals. The obtained sera were sampled and stored at − 20 °C.

A total of 40 μg of the protein extract was loaded on 12% SDS-PAGE, stained with (Coomassie Blue R) or transferred to Hybond ECL membrane (GE Healthcare) as described by Lima and colleagues (Lima Pde et al. 2015). Blocked membranes were incubated with anti-SidA polyclonal antibodies diluted 1:150 for 2 h. After incubation, the membrane was washed and incubated with anti-mouse secondary antibody alkaline phosphatase conjugated (1:20000). The reaction was developed using 5-bromo-4-chloro-3-indolylphosphate/nitroblue tetrazolium (BCIP/NBT). As a load control was employed anti-enolase polyclonal antibodies (Nogueira et al. 2010). The pixel intensity of the bands was analyzed using the ImageJ 1.51 software (Schneider et al. 2012) and expressed as arbitrary units.

### Extraction and digestion of proteins for nano-ESI-UPLC-MS^E^ acquisition

Protein extraction was performed by protocol described by Baeza and coleagues (Baeza et al. 2017). The protein concentration in the supernatant was determined using the Bradford reagent (Bradford 1976). Bovine serum albumin was used as a standard. Integrity of the proteins was verified using a 12% SDS-PAGE. A total of 150 μg of cytoplasmic protein was prepared for nanoUPLC-MS^E^ analysis, as previously described (Lima Pde et al. 2015; Murad et al. 2011). After digestion, the peptides were ressuspended in 30 μl of ultrapure water and subsequently purified in ZipTip C18 Pipette Tips (Millipore, MA, USA) and dried in a speed vacuum. The obtained peptides were suspended in 80 μl of a solution containing 20 mM of ammonium formiate and 200 fmol/μL of PHB (MassPREP™ protein). After solubilization, peptides were transferred to a Waters Total Recovery vial (Waters Corporation, MA, USA). For separation of tryptic peptides, Nanoscale LC was performed using an ACQUITY UPLC® M-Class system (Waters Corporation, MA, USA) (Tomazett et al. 2019).

### Data processing and protein identification

Data processing were performed as previously described (Lima Pde et al. 2015). In brief, for proteomic analyzes of the data obtained from the LC-MS^E^, the ProteinLynx Global Server version 3.0.2 (Waters, Manchester, UK) was employed. The processed spectra were searched against *P. brasiliensis* (*Pb*18) protein sequences (https://www.uniprot.org/proteomes/). The protein identification criteria also included the detection of at least 2 fragment ions per peptide, 5 fragments per protein and the determination of at least 1 peptide per protein. A protein that showed a variance coefficient of 0.057 and that was detected in all replicates was used to normalize the protein expression levels in the samples (PADG_04570). Expression^E^ informatics v.3.0.2 was used for quantitative comparisons. The mathematical model used to calculate the ratios was part of the Expression^E^ algorithm inside the PLGS software from the Waters Corporation (Geromanos et al. 2009). The minimum repeat rate for each protein in all replicates was 2. Protein tables generated by ProteinLynx Global Server were merged, and the dynamic range of the experiment was calculated using the software program MassPivot v1.0.1. The data obtained by NanoUPLC-MS^E^ were subjected to in silico analysis to identify functional classification. For this analysis, it was used FungiDBdatabase (https://fungidb.org/fungidb/).

### Construction and characterization of the *P. brasiliensis* SidA antisense-RNA strain

The functional evaluation of SidA was performed by antisense RNA technique, as described by Bailão and colleagues (Bailao et al. 2014) and Parente-Rocha et al. (2015). *A. tumefaciens* strain LBA 1100 was used for *P. brasiliensis* genetic transformation experiments. The cells from *A. tumefaciens* were cultured in induction medium (IM) for co-cultivation. Transformants were selected in BHI medium containing hygromycin 75 μg/mL (w/v) and randomly selected clones were confirmed for silencing by qRT- PCR. RNA extraction, cDNA synthesis and real-time PCR procedure were performed.

*P. brasiliensis* wild type (WT), empty vector (EV) and silenced for *sidA* gene (*AsSidA*) were cultured in liquid MMcM at 36 °C and 180 rpm in the presence of iron. Cell growth was evaluated by optical density at a wavelength of 600 nm every 24 h. Cell viability analysis was evaluated by staining with 1 μg/mL (wt/vol) propidium iodide (Sigma Aldrich). The samples were analyzed in a fluorescence microscope (Zeiss Axiocam MRc – Scope A1) (Zambuzzi-Carvalho et al. 2013).

### Siderophore production assayed by chrome azurol (CAS)

Siderophore production was analyzed as described previously (Silva-Bailao et al. 2014). For the overlay CAS (O-CAS) assay, 10^6^ yeast cells were grown in solid MMcM medium with no iron supplementation for 5 days. After that, 15 ml of CAS solution (Schwyn and Neilands 1987) were applied over the plates. The ternary complex Chrome Azurol S/Fe^3+^/hexadecyltrimetyl ammonium bromide (HDTMA) acts as an indicator of siderophore production, since it is originally blue and turns orange in presence of siderophores (Perez-Miranda et al. 2007).

The percentage of siderophores production was also determined (Machuca and Milagres 2003). Briefly, 10^6^ yeast cells were grown in MMcM liquid medium with no iron supplementation or with different concentrations of ammonium ferrous sulfate (2.5 μm, 5 μm, 10 μm and 30 μm). After 5 days of growth, the culture supernatants were collected by centrifugation at 10,000 x *g* for 1 min and 400 μl were incubated with the same volume of CAS liquid medium (Schwyn and Neilands 1987). The reference sample was prepared by adding 400 μL of sterile MMcM without iron or with different iron concentrations to 400 μL of CAS liquid medium. After 1 h of incubation at room temperature in the dark, the absorbance at 630 nm (Ultraspec 2000 UV/Visible Spectrophotometer Pharmacia Biotech) was determined. The percentage of siderophore activity was calculated by subtracting the sample absorbance values from the reference according to the following formula [(Ar–As/Ar)] × 100, in which Ar means absorbance of reference and As absorbance of sample.

### Standardization of *Tenebrio molitor* larvae as an infection model for *P. brasiliensis*

*T. molitor* larvae were acquired from a local supplier and maintained in oatmeal diet until experimentation. *P. brasiliensis* yeast cells were cultured in BHI as previously described, collected through centrifugation, washed three times with PBS 1X and resuspended in PBS. The cells were repeatedly passed through a 18-gauge needle coupled to a 5 mL syringe and later strained through a 40 μm nylon filter to obtain a homogenous cell suspension. The cells were counted with a hemocytometer and diluted to specific concentrations with PBS. Hemocoel injection was performed with a Hamilton syringe (Sigma-Aldrich) in *T. molitor* larvae weighing between 150 and 200 mg, showing light and uniform color and absence of pigmented spots. The infection was performed in the ventral part of the larva, in the second segment after the paws and the volume of cell suspension was 5 μL (e.g., harboring 1 × 10^5^, 1 × 10^6^ or 2 × 10^6^ cells). The larvae were kept in Petri dishes at 37 °C. The number of dead larvae was evaluated every 24 h for 10 days and deceased larvae were removed from the plate. As a control, the larvae were inoculated under the same conditions described above with PBS 1X. The experiments were performed with a total of 30 larvae per group.

### Evaluation of *AsSidA* virulence in *T. molitor* larvae

*P. brasiliensis* yeast cells of WT, EV and *AsSidA* mutants were cultured and prepared as previously described. The infection was performed as previously described, employing 2 × 10^6^ cells. The larvae were kept in Petri plates at 37 °C and the amount of dead larvae was evaluated every 24 h for 10 days. As a control, the larvae were inoculated under the same conditions described above with PBS 1X. The experiments were performed with a total of 30 larvae per group.

### Statistical analysis

Student’s *t*-test was used for the statistical analysis of the following experiments, enzymatic activity, qRT-PCR, immunobloting analysis and siderophores production. The following *P* values: *p* ≤ 0.0005, *p* ≤ 0.005 and *p* ≤ 0.05 were considered statistically significant, for each cited experiments, respectively. For the experiments in *T. molitor* the GraphPad Prism 5 program was used to generate the survival curve (using the Kaplan – Meier method) and for statistical analysis (Log-rank [Mantel-Cox]). *p* value < 0.05 was considered significant (de Souza et al. 2015; de Souza et al. 2018a, 2018b).

## RESULTS

### Molecular modeling of SidA demonstrates its interaction with substrates for the first step in siderophore biosynthesis

Although *P. brasiliensis* has orthologs for the all the components for siderophore biosynthesis, the functionality of this pathway still remains elusive. Therefore, we employ molecular modeling to describe in silico the structural characteristics of SidA and its possible ligands. The described SidA proteins harbor a conserved domain of the superfamily of oxygenases (Pfam 13,434; This domain is conserved in the putative SidA of *P. brasiliensis*) and require NADPH and FAD as cofactors. The crystallized protein with the most similar three-dimensional structure corresponds to *A. fumigatus* SidA, with 47% identity (PDBID: 4B63) (Franceschini et al. 2012; Krithika et al. 2006). Thus, molecular modeling of *P. brasiliensis* putative SidA was employed, in order to support and identify amino acid residues that hypothetically bind to FAD, NADPH and L-ornithine. The molecular modeling and three-dimensional structure comparison was based on *A. fumigatus* SidA (i.e, already crystallized protein; PDBID: 4B63) displaying 47% identity with *P. brasiliensis* putative SidA ortholog.

The (Additional file 2: Fig. S1 A) shows the alignment of *A. fumigatus* SidA (gray) and *P. brasiliensis* SidA (blue) showing a preserved site of interaction with L-ornithine, FAD and NADPH. The amino acid residues that interact with L-ornithine in *A. fumigatus* are LYS107, ASN323 and SER469, which in the *P. brasiliensis* model correspond to LYS88, ASN306 and SER448, respectively (Additional file 2: Fig. S1 B). The interaction between NADPH and FAD occurs through a triad of amino acids GLN102, VAL168 and ARG279 in the crystal of *A. fumigatus*, corresponding to GLN83, VAL149 and LYS262 in *P. brasiliensis* (Additional file 2: Fig. S1 C).

*P. brasiliensis* SidA was subjected to molecular dynamics (MD) simulation and the Clusters and RMSD analyzes showed that the equilibration phase started in 20 ns and stabilized in approximately 60 ns (Additional file 3: Fig. S2 A and B). Cluster 1 was the most relevant, as it remained between 20 ns until the end of the simulation, with no significant differences between the conformational models during this simulation period.

The quality parameters generated through clashscore and MolProbity showed high values for the model prior to MD 12.28 and 3.18, respectively. After the MD, the values were 0 and 1.28, respectively, showing a significant improvement in the quality of the structure, mainly considering the reduction of shocks between the atoms (Additional file 4: Table S2). In the Ramachandran diagrams (Additional file 3: Fig. S2 C and D) we observed a decrease in amino acid residues in non-permitted regions (according to the *phi* and *psi* angles).

The most flexible regions of *P. brasiliensis* SidA, according to the RMSF analysis, and the SidA pockets are shown (Additional file 3: Fig. S2 E and F). It is worth mentioning that the unstable regions highlighted in the RMSF do not correspond to the regions of the catalytic site. As the structural analyzes showed a high-quality for *P. brasiliensis* SidA model, this structure could be used for other structure-based approaches. Thus, the analysis of the molecular structure, suggests that SidA binds to L-ornithine as a substrate, and this reaction depends on NADPH and FAD.

### Expression and enzyme activity of SidA upon FOB exposure

**Fig. 1:**
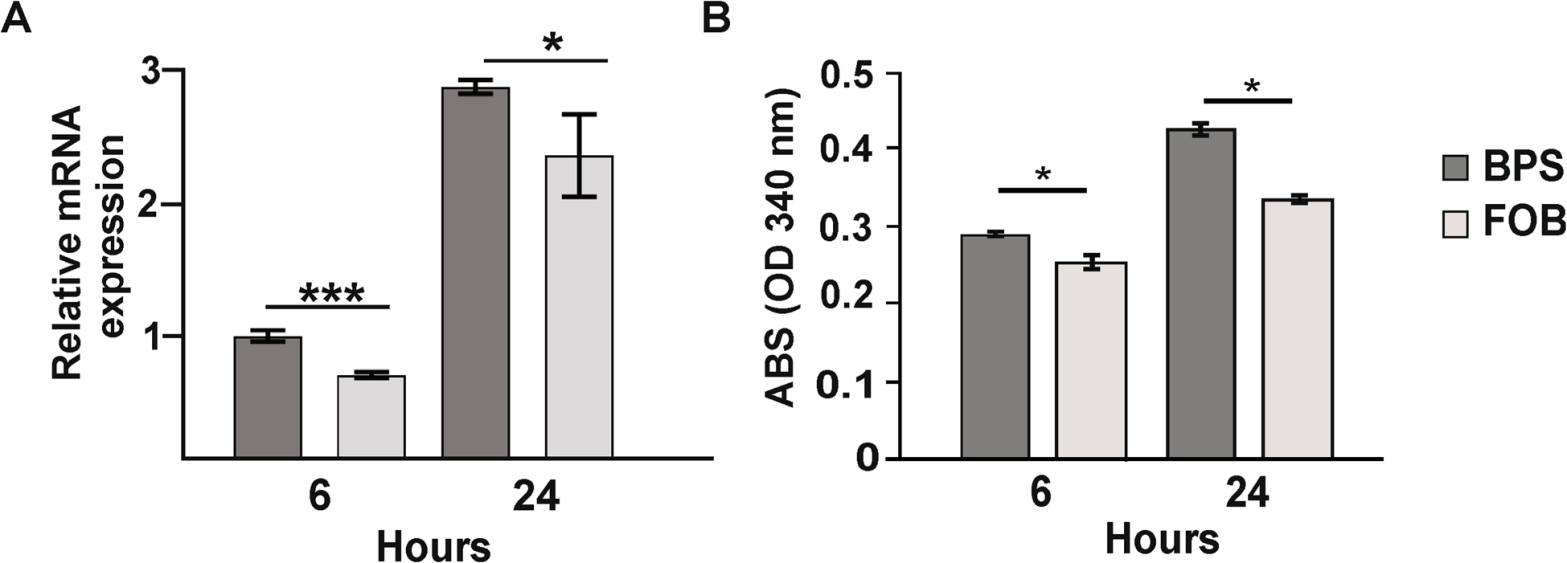
Expression of the transcript and enzymatic activity of *P. brasiliensis* SidA. **a** Relative expression of the *sidA* was analyzed in *P. brasiliensis* yeast cells grown for 6 and 24 h in presence of FOB (10 μM) or BPS (50 μM). Data were normalized to the gene encoding the 28 kDa ribonucleoprotein (GenBank XP_015701336). **b** To determine expression in the presence of FOB the enzymatic activity of SidA (L-ornithine-N^5^-oxygenase) was evaluated after growth of *P. brasiliensis* yeast cells in the presence of FOB or BPS. Results were obtained by absorbance at 340 nm. Student’s t test was used for statistical comparisons in both assays. Error bars represent standard deviation of three experimental replicates while *** demonstrate *p* ≤ 0.0005 and * represents *p* ≤ 0.05

Once the functionally of *P. brasiliensis* SidA was supported through molecular modelling, the effects of FOB treatment over *P. brasiliensis* was thorough examined. The transcript levels of *sidA* were evaluated in the presence of FOB. Notably, *sidA* transcripts were down regulated at 6 and 24 h in FOB condition when compared to to iron starvation condition (BPS) (Fig. 1a). Additionally, the enzymatic activity of SidA decreased in *P. brasiliensis* yeast cells grown in the presence of FOB (iron loaded siderophore) (Fig. 1b), poiting for a correlation between transcript levels and enzyme amount. The data strongly suggested that the presence of an exogenous siderophore represses the biosynthesis of endogenous siderophores, since the fungus can be using Fe^3+^ attached to the xenosiderophore as an alternative iron source.

### SidA recombinant protein and polyclonal antibodies production allows the identification of SidA in yeast cells

**Fig. 2:**
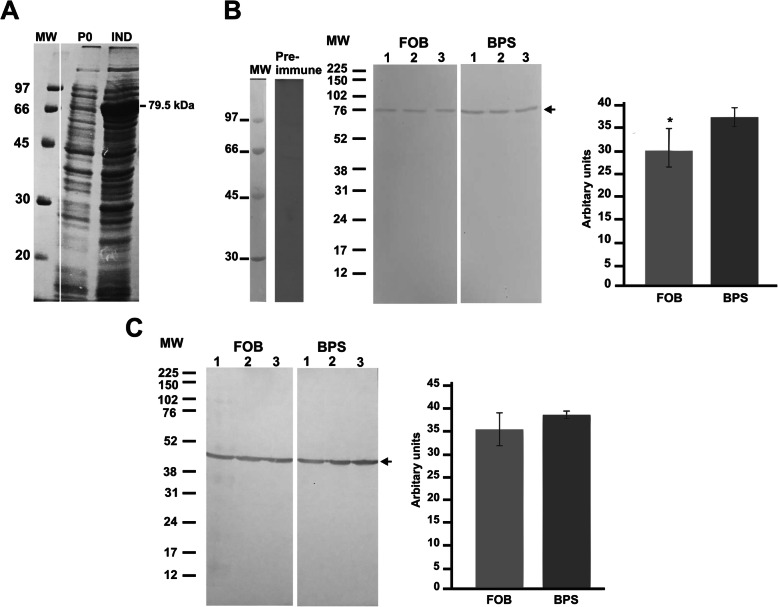
Expression of the recombinant protein SidA and immunoblotting analysis. **a** SDS-PAGE of SidA expression by *E. coli* Rosetta (DE3). 79.5 kDa corresponds to 53.5 kDa of SidA fused to 26 kDa of GST-tag. **b** Membranes containing extracts of cytoplasmic proteins of *P. brasiliensis* cultivated in FOB or BPS for 24 h were incubated with pre-immune sera and anti-*Pb*SidA polyclonal antibodies. The native SidA protein presents a molecular mass of 53.5 kDa (black arrow). **c** Membranes with the same extracts described above were incubated with anti-*Pb*enolase polyclonal antibodies. The pixel intensity of the immunoblotting bands was analyzed using the ImageJ 1.51 software and expressed as arbitrary units. The Student’s t test was used for statistical analysis. Error bars represent standard deviation of the experimental triplicate* *p* ≤ 0.05. MW: molecular weight marker. P0: *E. coli* before IPTG induction. IND: *E. coli* after IPTG induction (1 mM IPTG for 2 h)

The levels of SidA in *P. brasiliensis* cells were evaluated by western blotting with antibodies raised against recombinant SidA (rSidA). *P. brasiliensis* SidA protein was expressed in *E. coli* Rosetta (DE3) and rendered a protein with molecular mass of 79.5 kDa, corresponding to 53.5 kDa of SidA fused to 26 kDa of GST-tag (Fig. 2a). Following induction with IPTG, polyacrylamide gel pieces containing the recombinant protein were subjected to in-gel protein digestion for identification by LC-MS/MS approach; that confirmed the recombinant protein as SidA (Additional file 5: Fig. S3 and Additional file 6: Table S3). Immunoblotting results showed a decreased level of SidA in FOB-treated yeast cells when compared to BPS (Fig. 2b), corroborating the results obtained at transcript level. For loading control, membranes containing the same samples as in B were incubated with anti-*Pb*enolase polyclonal antibodies (Fig. 2c).

### Label free proteomic analysis reveals that SidA is repressed in yeast cells grown in the presence of FOB

**Fig. 3:**
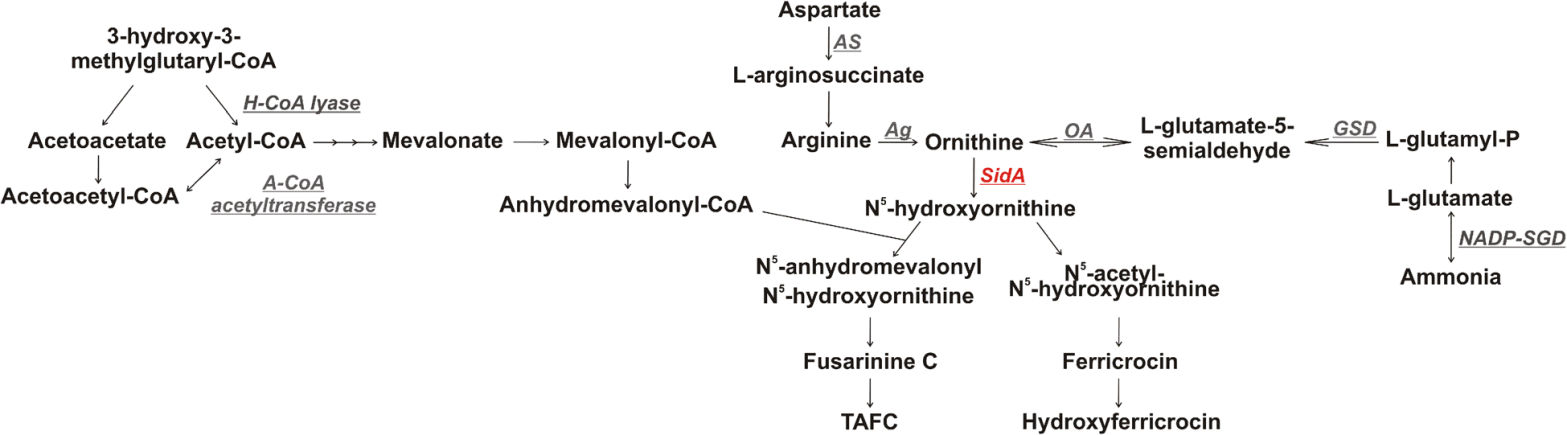
Proteins related to siderophore synthesis identified in the proteome. AS**:** Argininosuccinate synthase (PADG_00888). Ag: Arginase (PADG_00637). OA: Ornithine aminotransferase (PADG_01328). GSD: Glutamate-5-semialdehyde dehydrogenase (PADG_05337). NADP-SGD: NADP-specific glutamate dehydrogenase (PADG_04516). H-CoA L: Hydroxymethylglutaryl-CoA lyase (PADG_07031). A-CoA AT: Acetyl-CoA acetyltransferase (PADG_02751). SidA: L-ornithine-N^5^-monooxigenase (PADG_00097). The red color represents the repressed enzyme

Proteomic analysis of *P. brasiliensis* yeast cells in presence of FOB and BPS was performed to evaluate alterations in the protein profile between both conditions. It was identified 431 and 475 proteins in BPS or FOB, respectively, in both time points, of 6 and 24 h (data not shown). Table 1 features the identified proteins, related to the synthesis of siderophores, which include those down and non-regulated. Proteins such as arginase (PADG_00637), ornithine aminotransferase (PADG_01328), glutamate-5-semialdehyde dehydrogenase (PADG_05337), NADP-specific glutamate dehydrogenase (PADG_04516), arginosuccinate synthase (PADG_00888), hydroxymethylglutaryl-CoA lyase (PADG_07031) and acetyl-CoA acetyltransferase (PADG_2751), which are related to the synthesis of ornithine, arginine and acetyl-CoA, respectively, were identified in the proteome. Notably, only SidA was down regulated after 24 h of incubation with FOB (Fig. 3 and Table 1).

**Table 1:** Proteins related to the biosynthesis of siderophores identified in proteomic analysis

Accession number^**a**^	Description^**b**^	Time Point^**c**^	Score^**d**^
PADG_00637	Arginase (325 aa)	6 h	1131.29
PADG_00637	Arginase (325 aa)	24 h	1171.88
PADG_01328	Ornithine aminotransferase (461 aa)	6 h	2299.86
PADG_01328	Ornithine aminotransferase (461 aa)	24 h	1175.4
PADG_00888	Argininosuccinate synthase (416 aa)	6 h	3507.13
PADG_00888	Argininosuccinate synthase (416 aa)	24 h	4840.6
PADG_07031	Hydroxymethylglutaryl-CoA lyase (357 aa)	6 h	2406.52
PADG_07031	Hydroxymethylglutaryl-CoA lyase (357 aa)	24 h	2627.2
PADG_05337	Glutamate-5-semialdehyde dehydrogenase (457 aa)	6 h	1209.7
PADG_05337	Glutamate-5-semialdehyde dehydrogenase (457 aa)	24 h	771.05
PADG_04516	NADP-specific glutamate dehydrogenase (460 aa)	24 h	501.85
PADG_02751	Acetyl-CoA acetyltransferase (400 aa)	6 h	4328.06
PADG_02751	Acetyl-CoA acetyltransferase (400 aa)	24 h	3019.16
PADG_00097	L-ornithine-N^5^-monooxigenase (475 aa)^e^	24 h	360.99

^a, b^ Accession number and description of protein according to database of *Paracoccidioides* spp.(http://www.uniprot.proteomes/)

^c^ Time of treatment with FOB

^d^ Protein score obtained from MS data using the PLGS

^e^ Regulated Protein

### Characterization of *sidA* silenced strains

**Fig. 4:**
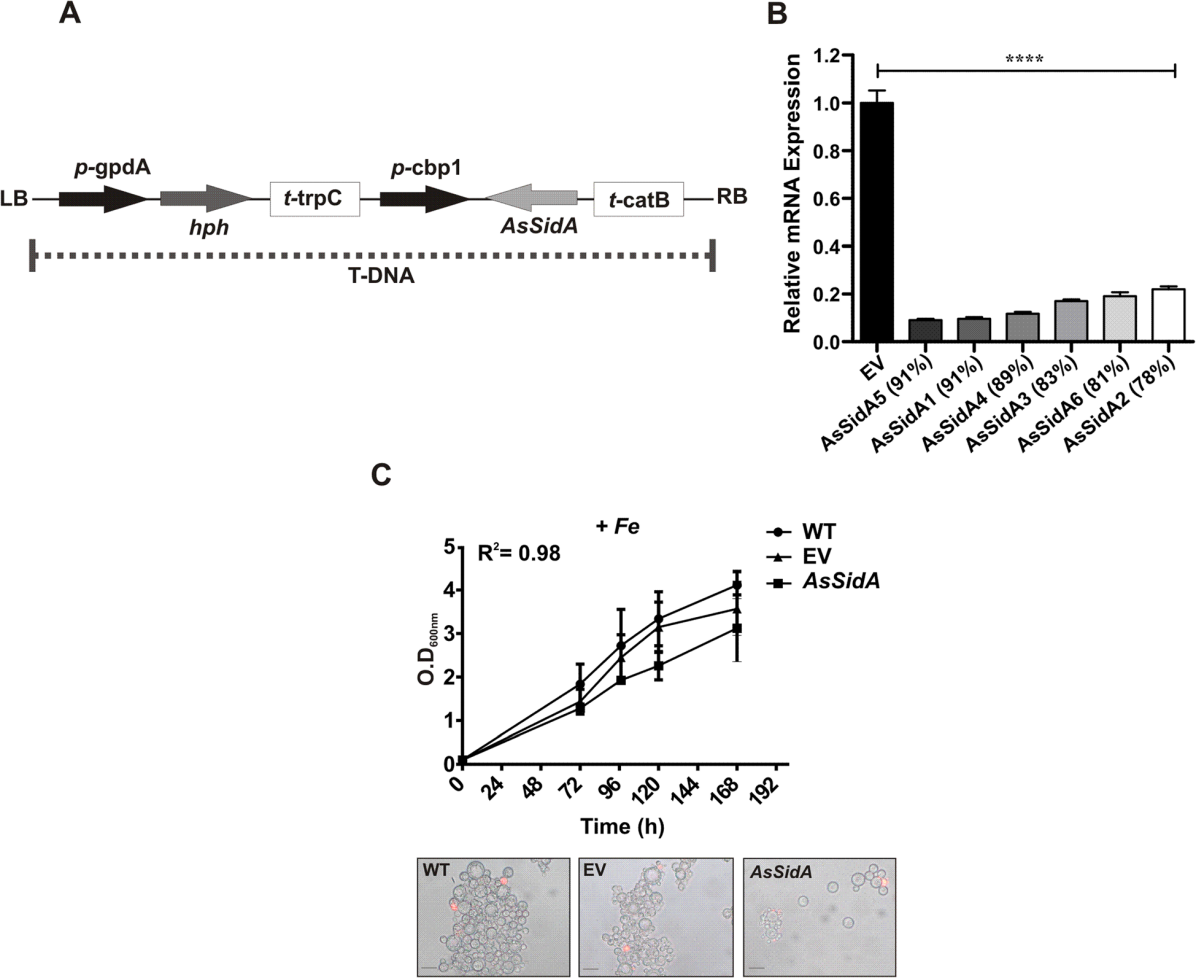
aRNA silencing of *sidA* gene via *A. tumefaciens*-mediated transformation (ATMT) in *P. brasiliensis*. **a** Schematic representation of the T-DNA cassette. The cassette is as following: *sidA* antisense fragment was placed under control of the calcium binding protein (*p*-cbp1) promoter of *H. capsulatum* and the terminator (*t*-catB) of *A. fumigatus*. The selection marker, was hygromycin B phosphotransferase (HPH), under control of glyceraldehyde 3-phosphate dehydrogenase of *A. nidulans* (*p*-gpdA) with the terminator (*t*-trpC) of *A. nidulans.***b** Relative quantification performed by qRT-PCR to confirm the gene silencing level in clones transformed with *Pb*SidA-aRNA. The transcript level of *Pb*WT transformed with the empty vector (EV) was also quantified and used as control. The actin gene (XP_010761942) was used as the endogenous control. The Student’s t-test was used for statistical comparisons. **** *p* < 0.0005. **c** Growth and viability of *P. brasiliensis* WT, EV and *AsSidA* strains. Yeasts strains were cultivated in McMM supplemented with 30 μM FeSO_4_ at 37 °C for 192 h. Growth curve profiles were determined by optical density, at a wavelength of 660 nm. Viability was accessed by staining of yeast cells with propidium iodide at 1 μg/ml on the last day of the growth curve. The images were obtained using an Axioscope A1 microscope (Carl Zeiss AG, Germany) and photographed at 493/636 nm

The data obtained from gene expression, enzymatic activity, proteome and molecular modeling of SidA point to the function of this protein in *P. brasiliensis* as an enzyme directly involved with the production of siderophores. Therefore, knockdown strains were constructed to determine the SidA role in *P. brasiliensis*. For that, it was used the antisense RNA technology, Fig. 4a depicts the *sidA* knockdown T-DNA cassette. This methodological approach provided *sidA*-knocked-down strains in *P. brasiliensis* yeast cells as demonstrated by qRT-PCR (Fig. 4b). The silencing percentage of six randomly selected clones ranged from 78 to 91% compared to those transformed with empty vector. *AsSidA* mutants grow similarly to the wild type and empty vector clones in MMcM medium supplemented with iron until 192 h. In addition, cells remained viable during the entire time growth (Fig. 4c).

### *sidA* silenced strains present reduced siderophore production

**Fig. 5:**
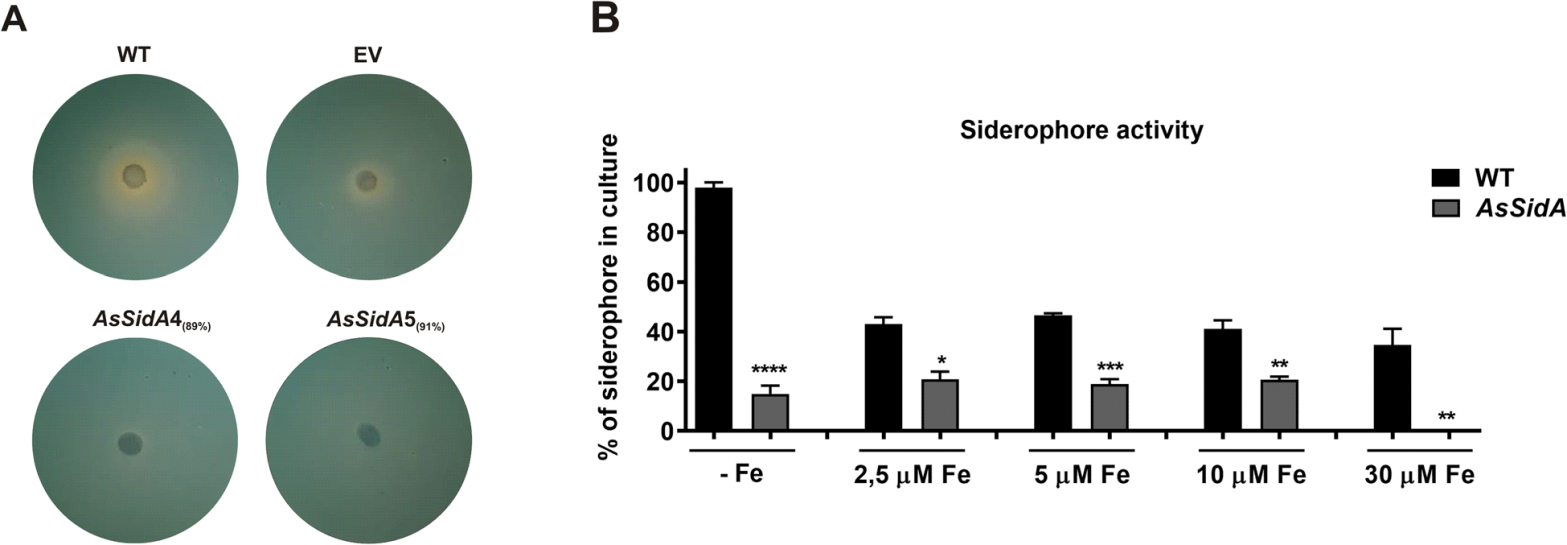
Production of siderophores in *sidA* silenced strains. **a** Wild type (WT), empty vector (EV) and silenced sidA (*AsSid4* and *AsSid5*) strains were grown in iron limited MMcM for 5 days. Following, the O-CAS plate assay was performed. The presence of secreted siderophores induces the formation of an orange halo. **b** After 5 days of growth in iron limited MMcM liquid medium or in MMcM added of different concentrations of inorganic iron, the percentage of siderophores produced by different strains was verified by the CAS liquid assay. Control of the reaction was performed by incubating the culture medium with CAS without fungal cells. The presence of siderophores was compared spectrophotometrically with the values obtained in the control by the values obtained from the supernatant of the different cultures. The results were presented in percentage. Statistical analysis was performed by Student’s t test. Error bars represent standard deviation of three experimental replicates while *** demonstrates *p* ≤ 0.0005, ** *p* ≤ 0.005 and * *p* ≤ 0.05

Silenced clones were subjected to iron deprivation and subsequently subjected to O-CAS assay. As expected, in iron scarcity, WT and EV strains are still able to produce siderophores, while theknockdown strains (*AsSidA4* and *AsSidA5*) displayed a reduced production of these compounds (Fig. 5a). Furthermore, the siderophore production was evaluated in WT and *AsSidA5* strains after 5 days of growth in iron deprivation and in different iron concentrations (Fig. 5b). There was a pronounced decrease in siderophores production in *AsSidA5* strain in presence and absence of iron.

### *SidA* can be a putative virulence factor in *P. brasiliensis*

**Fig. 6:**
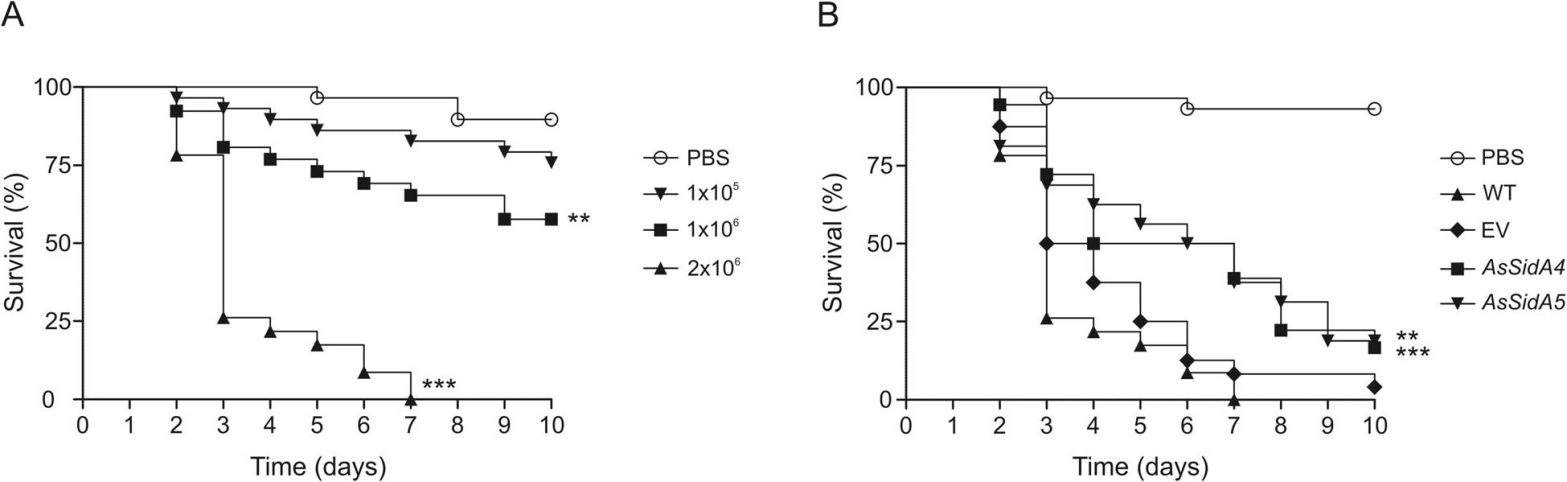
Survival curves of *T. molitor* infected with *P. brasiliensis* yeast strains. *T. molitor* larvae were infected with different concentrations of *P. brasiliensis* yeast cells of WT strain (**a**) and with 2 × 10^6^ yeast cells of the WT, EV and *AsSidA* mutant strains (*AsSidA4* and *AsSidA5*) (**b**). As a control, the larvae were inoculated with PBS only. The experiments were carried with a total of 30 larvae for each condition analyzed in a period of 10 days. The survival of *T. molitor* larvae is shown as a percentage. (*** *p*< 0.0005, ** *p*< 0.005 and * *p*< 0.05)

Since it was the first virulence study of the effects of *P. brasiliensis* over *T. molitor* through intra-hemocoel injection, it was necessary to standardize the amount of cells needed to accomplish a successful infection in the larvae. First, only yeast cells of WT strain were employed to perform the standardization, followed by mutant analysis (EV and *AsSidA* strains). Thus, different concentrations of *P. brasiliensis* cells (1 × 10^5^, 1 × 10^6^ or 2 × 10^6^) were inoculated in *T. molitor* larvae. As expected, larvae inoculated with *P. brasiliensis* cells showed an increase in the mortality rate as the fungus concentration also increased. The concentrations of 1 × 10^5^ cells was not enough to kill all *T. molitor* larvae, which showed a survival rate of 75% after 10 days of infection. Furthermore, 1 × 10^6^ cells of *P. brasiliensis* were not able to kill all larvae, presenting a survival rate of 57% after 10 days. In light of these results, an ideal concentration of 2 × 10^6^ cells was chosen. This concentration led to 100% mortality after 7 days post infection (Fig. 6a).

Once the ideal cell concentration was established, the ability of the *AsSidA* strains (*AsSidA4* and *AsSidA5*) to kill the *T. molitor* larvae was evaluated, comparing with WT and EV strains (Fig. 6b). Larvae infected with EV had a survival rate of only 4% after 10 days of infection, while larvae infected with *AsSidA* mutant strains had a statistically significant reduction in larvae mortality, with a survival rate of 16 and 18%, respectively. These results point that the silencing of *SidA* affects the pathogenesis of *P. brasiliensis*, further supporting the role of siderophores on *Paracoccidioides* infection process.

## DISCUSSION

SidA is an enzyme specific for hydroxylation of ornithine, a precursor molecule of ferricrome siderophores biosynthesis (Chocklett and Sobrado 2010). The three-dimensional structure of SidA in *A. fumigatus* demonstrates the presence of amino acid residues essential for both NAPDH association and ornithine binding and hydroxylation (Robinson et al. 2015; Robinson et al. 2014). In this sense, SidA structure from *P. brasiliensis* was studied since the capacity of ornithine hydroxylation and use of NADPH as cofactor are specific to this enzyme (Chocklett and Sobrado 2010), which are influenced by the amino acids present in its structure (Robinson et al. 2014; Robinson et al. 2015). The characterization of the three-dimensional SidA structure of *P. brasiliensis* was performed by molecular modeling based on *A. fumigatus* crystal, which is most similar structure available (Sanchez et al. 2017). The amino acids from the active site ASN, SER and LYS coordinate L-ornithine in the pocket so that it can interact with NADPH and FAD. In *A. fumigatus*, mutations targeting residues in the L-ornithine binding site altered the substrate coupling and the kinetic parameters of the reaction. Among all mutations analyzed, alterations in LYS107, which corresponds to LYS88 in *P. brasiliensis*, led to important changes in the enzymatic kinetics and, therefore, it was considered the most important amino acid involved in enzymatic kinetics (Kosman 2003). Thus, molecular structure data confirm that the active site of *P. brasiliensis* SidA is conserved and has similar interaction residues when compared to *A. fumigatus* SidA.

We have previously demonstrated that members of the *Paracoccidioides* complex can use the heterologous siderophore ferrioxamine B (FOB) as an iron source and produce siderophores when grown in iron starvation conditions (Silva-Bailao et al. 2014). In an attempt to investigate the connection between siderophore biosynthesis and FOB utilization, experiments were conducted to demonstrate the repression of *sidA* when *P. brasiliensis* was incubated with FOB. In addition, SidA accumulation, at both transcriptional and proteomic level, and the cognate enzymatic activity were reduced in cells cultured in the presence of FOB, when compared to cells growing in iron depleted medium. Similar results employing xenosiderophores were described for *Candida albicans, Saccharomyces cerevisiae* and *A. nidulans* (Heymann et al. 2002; Haas 2003; Philpott and Protchenko 2008).

In order to better explore the impact of FOB over *P. brasiliensis* growth a proteomic analysis was performed. Data allowed the identification of several proteins involved in siderophores biosynthesis. Notably, the decrease of SidA levels in presence of FOB was the most glaring result. Thus, all the data obtained in this study demonstrate that, in the presence of FOB, SidA is repressed both transcriptionally and translationally, and evidences the siderophore biosynthesis pathway in *P. brasiliensis.*

To confirm the function of SidA in the production of siderophores and the role of this protein in the biology of *P. brasiliensis*, we silenced the *sidA* gene by using antisense RNA technique. The same system was already reported to obtain *P. brasiliensis* silenced clones for proteins playing relevant roles in the fungal pathobiology, e.g., Cdc42p (Almeida et al. 2009), *Pb*HAD32 (Hernandez et al. 2010), *Pb*Rbt5 (Bailao et al. 2014), *Pb*ccp (Parente-Rocha et al. 2015), *Pb*14–3-3 (Marcos et al. 2016), and *Pb*PCN (Fernandes et al. 2017). Here we have successfully obtained knockdown strains for *PbsidA*, as demonstrated by qRT-PCR.

As silencing of *sidA* gene did not impair fungal growth in regular culture media, the phenotypic characterization of this gene was performed employing iron deprivation conditions. The qualitative O-CAS assay showed that production of siderophores in the knockdown strains was diminished when compared to the WT and EV strains. Furthermore, a semi-quantitative CAS liquid assay also highlighted the diminished production of siderophores with and without iron.

Although murine models are valuable tools for in vivo infection studies, invertebrate models are becoming prominent alternatives. The ethical reasons linked to vertebrate models, as well as the high costs for animal management have propelled the examination of viable substitutes (Wilson-Sanders 2011). Several studies have demonstrated the use of *Galleria mellonella* larvae to characterize the infection process in pathogenic fungi, such as *Histoplasma capsulatum*, *C. albicans*, *Cryptococcus neoformans* and *P. brasiliensis* (Thomaz et al. 2013; Vargas et al. 2015; Bouklas et al. 2015; Scorzoni et al. 2015; Marcos et al. 2019). However, *G. mellonella* is an animal that requires almost daily handling, as well as these insects are not sold worldwide (Jorjao et al. 2018). An alternative that has been explored are larvae of *T. molitor*, an animal that is easy to handle, is marketed by several suppliers and is a low cost insect. Furthermore, the *T. molitor* larvae have been standardized as an in vivo infection model for several fungal pathogens as *C. albicans*, *C. neoformans*, *Malassezia furfur* and *Fonsecaea pedrosoi* (de Souza et al. 2015; Silva et al. 2018; Fornari et al. 2018). Here, we explored the application of *T. molitor* as an invertebrate infection model for *P. brasiliensis*. Noteworthy, increasing the viable cell concentration in the inoculum also increases *T. molitor* larvae mortality. The same observations can be draw for *C. albicans* and *C. neoformans*, demonstrating that this model is efficient for assessing the virulence of pathogenic fungi (de Souza et al. 2015).

Metal restriction is one of the several mechanisms employed by host cells to inhibit microbial development (Ganz 2016; Ganz 2018; Brechting and Rappleye 2019). Thus, the presence of a high affinity iron acquisition systems appears to be an essential strategy to circumvent the absence of this metal during growth in a hostile environment (McDonagh et al. 2008; Hilty et al. 2011; Pasricha et al. 2016; Kalidasan et al. 2018). In several fungal pathogens, siderophores are important for virulence and host-pathogen interaction (Heymann et al. 2002; Schrettl et al. 2004; Schrettl et al. 2010; Nevitt and Thiele 2011). For example, in *H. capsulatum*, the deletion of *sid1*, the homologue of *sidA* of *A. fumigatus*, promoted decreasing of siderophore production, resulting in diminished proliferation inside macrophages and murine pulmonary colonization (Hwang et al. 2008). Similarly, previous results pointed for a putative role of these molecules in pathogenesis of *P. brasiliensis*, since the expression of sidA was highly induced during fungal infection to macrophages (Silva-Bailao et al. 2014). The bioassays conducted here with *AsSidA* mutants support a main role of siderophores in the infection process of *Paracoccidioides* species. *AsSidA* mutants presented reduced virulence to *T. molitor* larvae, reasserting the importance of iron for host colonization.

## CONCLUSIONS

Although previous results pointed that *P. brasiliensis* harbors a complete pathway for siderophore production several aspects about SidA regulation and activity were still to be determined. The molecular modelling, here employed, helped to highlight structural similarities between *A. fumigatus* SidA and *P. brasiliensis* SidA. Furthermore, the expression of SidA is repressed, at transcriptional and translational levels, in the presence of a xenosiderophore FOB. Moreover, *sidA* silencing in blocks the production of siderophores and promotes decrease in fungal virulence *P. brasiliensis*. Due to the relevance of iron for fungal survival inside the host, strategies aiming to block siderophore biosynthesis can be successful, helping to eliminate the infection. In this way, future studies will focus on molecules capable to block SidA activity.

## Supplementary information

**Additional file 1: Figure S1.** Molecular structure of L-ornithine-N^5^-monooxygenase. (A) *Af*SidA from *Aspergillus fumigatus* PDBID: 4B63 (gray) and *Pb*SidA (blue) alignment using the Pymol viewer, evidencing the interactions among L-ornithine, FAD and NADPH. (B) Amino acid residues of *P. brasiliensis* SidA described as mainly involved in the interaction with L-ornithine and (C) cofactors are essential for the maintenance of them in the active site.

**Additional file 2: Figure S2.** Molecular dynamics of *P. brasiliensis* SidA. (A) Cluster and (B) RMSD graphs. It is observed that the stability starts at approximately 20 ns and that the most representative conformational mode of the trajectory is cluster 1. (C) Ramachandran diagrams of the three-dimensional model of SidA before molecular dynamics and (D) after molecular dynamics. (E) RMSF graph showing the more flexible residues (red) along the molecular dynamics. (F**)** Three-dimensional structure of SidA showing the most flexible regions (red) and pockets of the active site (green and orange).

**Additional file 3: Figure S3.** Characterization of the recombinant protein. MS/MS spectrum of the recombinant protein SidA identified by mass spectrometry.

**Additional file 4: Table S1.** Oligonucleotides used in this study.

**Additional file 5: Table S2.** Analysis of the quality of the SidA models through the Molprobity server.

**Additional file 6: Table S3.**

## Data Availability

Not applicable.
