# Supplementary figures and images for: Molecular characterization of siderophore biosynthesis in Paracoccidioides brasiliensis
Source: IMA Fungus. 2020 Jun 29;11:11. doi: 10.1186/s43008-020-00035-x (PMC7359926; doi:10.1186/s43008-020-00035-x)

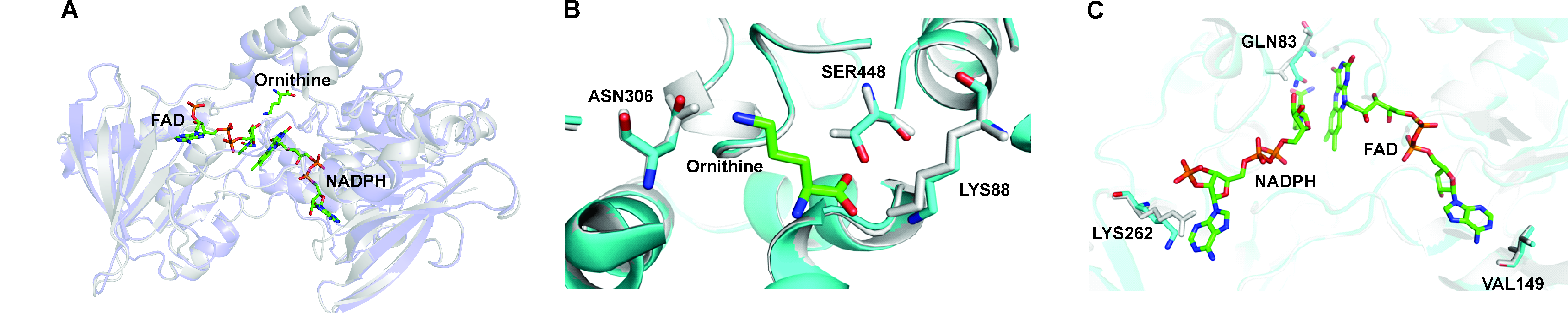

Supplement: Supplementary file 1 — Additional file 1: Figure S1. Molecular structure of L-ornithine-N5-monooxygenase. (A) AfSidA from Aspergillus fumigatus PDBID: 4B63 (gray) and PbSidA (blue) alignment using the Pymol viewer, evidencing the interactions among L-ornithine, FAD and NADPH. (B) Amino acid residues of P. brasiliensis SidA described as mainly involved in the interaction with L-ornithine and (C) cofactors are essential for the maintenance of them in the active site. [file 43008_2020_35_MOESM1_ESM.tif]

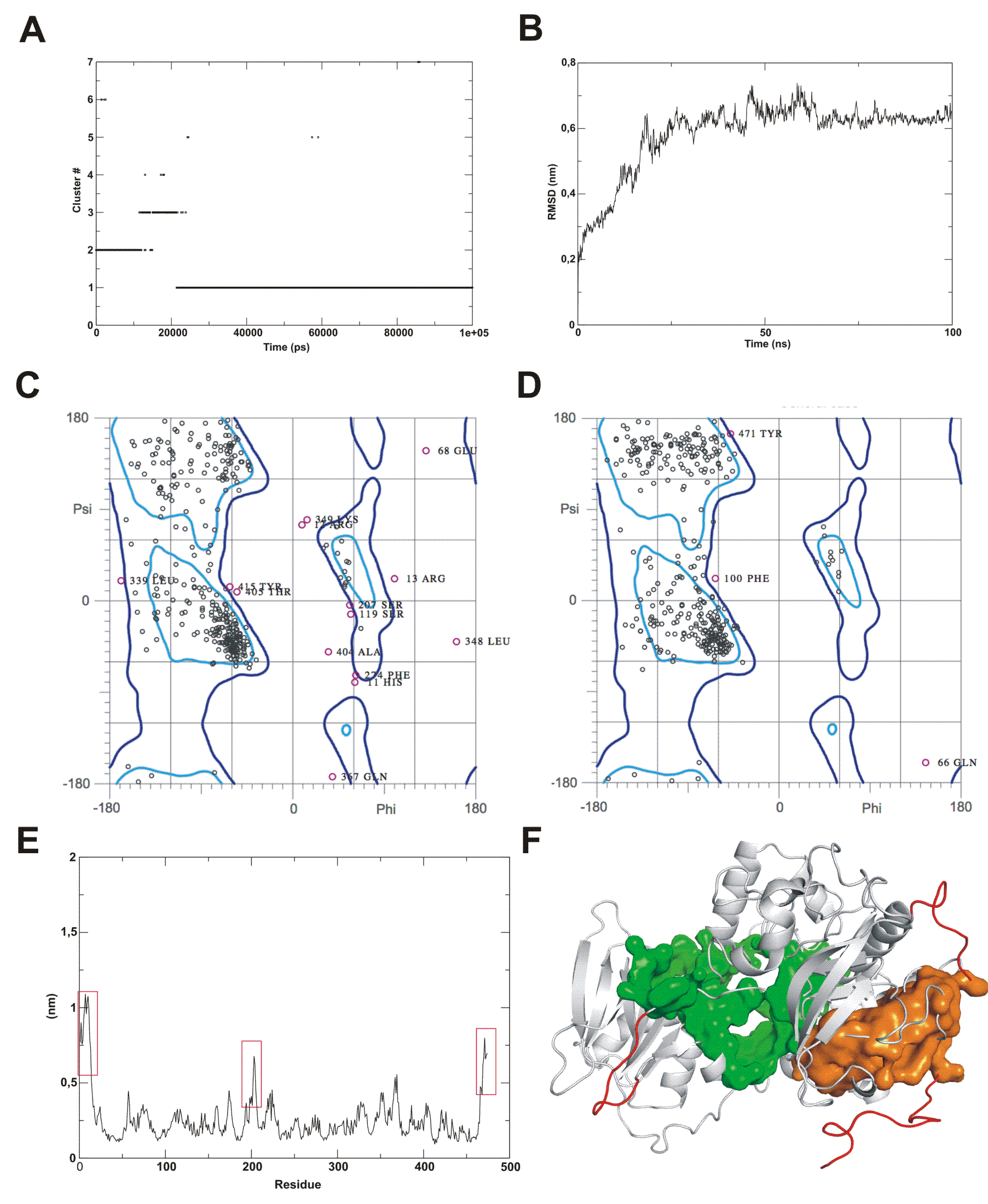

Supplement: Supplementary file 2 — Additional file 2: Figure S2. Molecular dynamics of P. brasiliensis SidA. (A) Cluster and (B) RMSD graphs. It is observed that the stability starts at approximately 20 ns and that the most representative conformational mode of the trajectory is cluster 1. (C) Ramachandran diagrams of the three-dimensional model of SidA before molecular dynamics and (D) after molecular dynamics. (E) RMSF graph showing the more flexible residues (red) along the molecular dynamics. (F) Three-dimensional structure of SidA showing the most flexible regions (red) and pockets of the active site (green and orange). [file 43008_2020_35_MOESM2_ESM.tif]

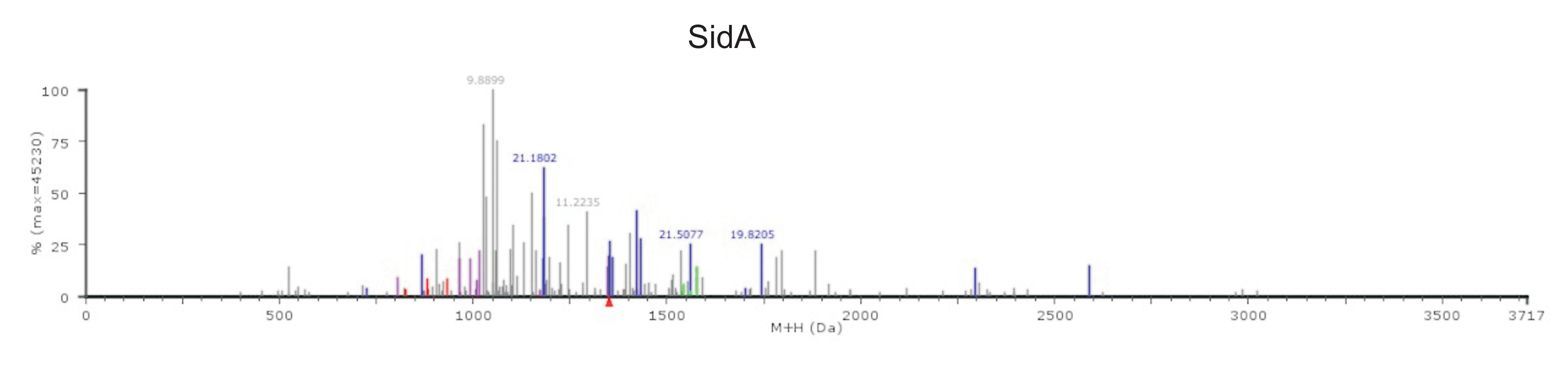

Supplement: Supplementary file 3 — Additional file 3: Figure S3. Characterization of the recombinant protein. MS/MS spectrum of the recombinant protein SidA identified by mass spectrometry. [file 43008_2020_35_MOESM3_ESM.tif]
