# Supplementary material for: Molecular characterization of siderophore biosynthesis in Paracoccidioides brasiliensis
Source: IMA Fungus. 2020 Jun 29;11:11. doi: 10.1186/s43008-020-00035-x (PMC7359926; doi:10.1186/s43008-020-00035-x)
Supplement: Supplementary file 4 — Additional file 4: Table S1. Oligonucleotides used in this study. [file 43008_2020_35_MOESM4_ESM.docx]

**Table S1 Oligonucleotides used in this study**

| Accession number^a^ | Sequence |
| --- | --- |
| sidA (PADG_00097) | Foward: 5’ GCGACGATAGCCCATTTGTC 3’  Reverse: 5’ATTAGCAGGATTAGGATCAAGG 3’ |
| Actin (XP_010761942) | Foward: 5’ CGTCCTCGCCATCATGGTAT 3’  Reverse: 5’ TCTCCATATCATCCCAGTTCG 3’ |
| 28 kDa ribonucleoportein (XP_015701336) | Foward: 5’ GAAAGGGTTCGGCTACGTTG 3’  Reverse: 5’ ATCTCTGGGGGCAGCATTTG 3’ |
| rSidA* (PADG_00097) | Foward:  5’ GGTTCCGCGTGGATCCATGGAGACCGTTATCAAGAA 3’  Reverse:  5’ GTCGACCCGGGAATTCCTATAGTCGTGCGTAATTCT 3’ |

^a^ Accession numbers according to database of *Paracoccidioides* spp. (https://blast.ncbi.nlm.nih.gov/Blast.cgi)

*Primers used for obtaining the recombinant SidA protein
