# Supplementary material for: Molecular characterization of siderophore biosynthesis in Paracoccidioides brasiliensis
Source: IMA Fungus. 2020 Jun 29;11:11. doi: 10.1186/s43008-020-00035-x (PMC7359926; doi:10.1186/s43008-020-00035-x)
Supplement: Supplementary file 5 — Additional file 5: Table S2. Analysis of the quality of the SidA models through the Molprobity server. [file 43008_2020_35_MOESM5_ESM.docx]

**Table S2** **Analysis of the quality of the SidA models through the Molprobity server.**

|  | SidA model before MD^*^ | SidA model after MD^*^ |
| --- | --- | --- |
| Clashscore | 12.28 | 0 |
| Molprobity score | 3.18 | 1.28 |
| Ramachandran outliers | 26 aa | 3 aa |

*Molecular dynamics
