# Supplementary material for: Molecular characterization of siderophore biosynthesis in Paracoccidioides brasiliensis
Source: IMA Fungus. 2020 Jun 29;11:11. doi: 10.1186/s43008-020-00035-x (PMC7359926; doi:10.1186/s43008-020-00035-x)
Supplement: Supplementary file 6 — Additional file 6: Table S3. [file 43008_2020_35_MOESM6_ESM.docx]

**Table S3 Identification of recombinant SidA by mass spectrometry.**

| Accession number^a^ | Description^b^ | mW (Da)^c^ | pI^d^ | Protein Score^e^ | Peptides^f^ | Peptides sequence^g^ | Coverage (%)^h^ |
| --- | --- | --- | --- | --- | --- | --- | --- |
| PADG_00097 | Hypothetical protein (475aa) | 53505 | 7.0854 | 1805.296 | 18 | RSSELLDSILASSKR  RYDVVVLATGYTRN  KFLDPNPANWSLRL  RLSLLESVYEKL  KELPNNQVELHVKD  KRAENYARL  REFGWHTGMLLPGSKM  KMQISFIKD  RELSNEPLPSTVIHSSVYLESEQKF  RELSAKH  RRVLFLERQ  KWCASHFDDWVQYKQ  RAENYARL  KDTQSGQIESSGERY  RERGGSYRF  KQEVLSVAAAEARPGWPAEHFKV  AENYARL  REFGWHTGMLLPGSKM | 44.3038 |

^a^ Available at NCBI database ([https://www.ncbi.nlm.nih.gov](https://www.ncbi.nlm.nih.gov/))

^b^ Protein annotation of the *Paracoccidioides* genome database.

^c^ Molecular Weight.

^d^ Isoelectric point.

^e^ Protein score obtained from MS data using the PLGS.

^f^  Number of peptides identified.

^g^ Sequence of identified peptides.

^h^ Average coverage.
